# Chloroplast Genome Analysis of Resurrection Tertiary Relict *Haberlea rhodopensis* Highlights Genes Important for Desiccation Stress Response

**DOI:** 10.3389/fpls.2017.00204

**Published:** 2017-02-20

**Authors:** Zdravka Ivanova, Gaurav Sablok, Evelina Daskalova, Gergana Zahmanova, Elena Apostolova, Galina Yahubyan, Vesselin Baev

**Affiliations:** ^1^Department of Plant Physiology and Molecular Biology, University of PlovdivPlovdiv, Bulgaria; ^2^Plant Functional Biology and Climate Change Cluster, University of Technology at Sydney, SydneyNSW, Australia

**Keywords:** *Haberlea rhodopensis*, desiccation stress, chloroplast genome, SSR, site-specific selection, *rbcL*

## Abstract

*Haberlea rhodopensis* is a paleolithic tertiary relict species, best known as a resurrection plant with remarkable tolerance to desiccation. When exposed to severe drought stress, *H. rhodopensis* shows an ability to maintain the structural integrity of its photosynthetic apparatus, which re-activates easily upon rehydration. We present here the results from the assembly and annotation of the chloroplast (cp) genome of *H. rhodopensis*, which was further subjected to comparative analysis with the cp genomes of closely related species. *H. rhodopensis* showed a cp genome size of 153,099 bp, harboring a pair of inverted repeats (IR) of 25,415 bp separated by small and large copy regions (SSC and LSC) of 17,826 and 84,443 bp. The genome structure, gene order, GC content and codon usage are similar to those of the typical angiosperm cp genomes. The genome hosts 137 genes representing 70.66% of the plastome, which includes 86 protein-coding genes, 36 tRNAs, and 4 rRNAs. A comparative plastome analysis with other closely related Lamiales members revealed conserved gene order in the IR and LSC/SSC regions. A phylogenetic analysis based on protein-coding genes from 33 species defines this species as belonging to the Gesneriaceae family. From an evolutionary point of view, a site-specific selection analysis detected positively selected sites in 17 genes, most of which are involved in photosynthesis (e.g., *rbcL, ndhF, accD, atpE*, etc.). The observed codon substitutions may be interpreted as being a consequence of molecular adaptation to drought stress, which ensures an evolutionary advantage to *H. rhodopensis.*

## Introduction

Chloroplasts are uniparentally inherited organelles in plant cells; they play an important role in many plant cell functions, including photosynthesis, carbon fixation, and stress response. In angiosperms, the chloroplast genome has a conserved quadripartite structure composed of two copies of inverted repeat (IR), one large single copy (LSC), and one small single copy (SSC) (Palmer, 1985). In contrast, extensive loss of the IR copies has been observed in gymnosperms (Wu et al., 2011). Although chloroplast shows evolutionary conservation across the tree of life, an accelerated rate of evolution has been widely observed in particular genes. For example, *rbcL*, which encodes the large subunit of ribulose-1,5-bisphosphate carboxylase/oxy-genase (RUBISCO) has been shown to play a fundamental role in light-dark state transitions (Morton and Clegg, 1993). It is worth mentioning that in addition to *rbcL*, chloroplast-encoded low molecular mass subunits of Photosystem II (PSII), including *psbI, psbJ, psbL, psbM*, and *psbTc* (Umate et al., 2008) and *psbA* mRNA translation (Kim and Mullet, 1994), are also under the influence of light transitions. Taking into account these interconnections, it can be assumed that chloroplast represents a major organelle that can be very important when studying the role of desiccation stress.

Genomic organization of plastome in photosynthetic plants comprises up to 88 protein coding genes and, in most eudicots, about 35 structural RNA genes, totaling 100–120 unique genes (Wicke et al., 2011). After the acquisition of chloroplasts, many genes relocated from the ancestral organellar genomes to the nucleus. As remodeled nuclear copies of organelle genes usurped the functions of those located in the organelle, biochemical pathways were transferred entirely from the chloroplasts to the cytosol and the plastid genomes were reduced in size. The relentless influx of organelle DNA into the nucleus has resulted in a decreased organelle autonomy and increased nuclear complexity (Timmis et al., 2004). In turn, the nucleus, depends on signals coming from the chloroplasts that transfer information to the nucleus via “retrograde signaling.” This allows modification of the nuclear gene expression according to the status of the chloroplast (Nott et al., 2006). Besides having a vital role in cellular communication, retrograde signaling plays an important role in the adaptive responses of plants to stress (Sun and Guo, 2016).

*Haberlea rhodopensis* Friv. belonging of the Gesneriaceae family is a homoiochlorophyllous plant that retains chlorophyll in a readily recoverable form throughout desiccation (Georgieva et al., 2005) and is a tertiary relic species, endemic to the Balkan peninsula. In addition to its homoiochlorophyllous nature, *H. rhodopensis* has the capability of resurrection (survival of extreme vegetative dehydration), a trait that is of significant importance in global climate change. Desiccation tolerance is one of the most widely described traits studied in this paleoendemic species, and previous studies have shown that light absorption and oxygen intake evolution play a key role in the adaptation of this species to desiccation stress (Heber et al., 2007; Heber, 2012). Drought resistance and rapid recovery of *H. rhodopensis* after rehydration were attributed to specific characteristics of the chloroplast of this species, including unchanged chlorophyll content, maintenance of chlorophyll–protein complexes, reversible modifications in PSII electron transport, and enhanced dissipation of non-radiative energy (Georgieva et al., 2007; Mihailova et al., 2011). From an evolutionary point of view, the origin of European Gesneriaceae genera has been dated back to the early Oligocene, while the *Haberlea* lineage emerged in the late Oligocene as suggested by population genetic (ISSRs) analyses of the chloroplast encoded *atpB-rbcL. trnH-psbA*, and *trnL-F* genes (Petrova et al., 2015). Despite its importance as a resurrection plant, there is a lack of studies using the chloroplast genome of *Haberlea* lineage to understand its molecular evolution and resolve the phylogenetic position of *H. rhodopensis* with respect to Lamiales.

In the present paper, we reconstruct the whole chloroplast genome by using next-generation sequencing and applying a combination of *de novo* and reference-guided assembly. This will help to delineate the phylogenetic position of this species and to understand the role of natural selection in the adaptation of *H. rhodopensis* to drought stress. In this study, we report on a 153,099 bp plastome of *H. rhodopensis*, analyze the genomic features and structure of its genome, and conduct comparative genomic studies to inform an improved understanding of the organelle genome evolution of this resurrection plant.

## Materials and Methods

### DNA Extraction and Sequencing

*Haberlea rhodopensis* samples were collected from Rhodopi mountain, Bulgaria (location 42°1′N 24°52′E). Chloroplast DNA was isolated from leaf tissue of 16 individual plants. For an optimal yield of intact chloroplasts, 40/80% Percoll gradient (Chloroplast Isolation Kit – Sigma-Aldrich) was used. Chloroplast DNA was extracted using DNeasy Plant Mini Kit (QIAGEN). Two biological replicates were performed. Library preparation and sequencing were performed at BGI-Shenzhen, China. For each replicate, the isolated DNA was used to generate 100-bp paired-end (PE) libraries with insert size of 170 bp, in accordance with the Illumina Hiseq2000 standard protocol. In our case, the 100 bp paired-end reads are overlapping approximately with 30 bp, thus producing longer (joined) fragments of about 170 bp, corresponding to the insert size. This method of joined pair-end reads was used as it increases the accuracy of the assembly.

### Genome Assembly

Prior to *de novo* genome assembly, raw reads were mapped to the NCBI Viridiplantae chloroplast genomes using BWA to filter the non-chloroplastic reads (Li and Durbin, 2010) in order to avoid contamination of mtDNA and nuclear DNA. Quality assessment of extracted chloroplast reads was performed by FASTX-Toolkit^1^. High-quality paired-end reads were merged by FLASH (Magoc and Salzberg, 2011). Length distribution and quality of the merged reads are presented in **Supplementary Data Sheet S1**. Subsequently merged reads were assembled using Abyss version 1.9.0 with k-mer = 55 in single-end mode. Assembly N50, L50 and related statistics were evaluated using QUAST (Gurevich et al., 2013) and assemblies with the most consistent results in terms of assembly evaluation parameters (N50 and L50) retained. QUAST results are shown in **Supplementary Data Sheet S1**. Assembled contigs were scaffolded using SSPACE 3.0 (Boetzer et al., 2011). Re-ordering of draft genome scaffolds based on comparison to the reference genome of *Boea hygrometrica* was performed using Abacas 1.3.1 (Assefa et al., 2009) and MUMmer (Kurtz et al., 2004).

Due to the limitations in the assembly process, a single fragment with twice the coverage is expected in the case of duplicated copies. After the identification, assembly, and scaffolding of such a fragment (a contig constituting an IR region), Abacas 1.3.1 was used to align, order and orientate all currently assembled contigs based on the reference plastome of *Boea hygrometrica*. The resulting output was a pseudomolecule of the draft chloroplast genome, where the IR region contig was represented twice. The key step in this standard approach is that a reference from a closely related species is used (e.g., *B. hygrometrica*), which ensures that the IR regions are accurately identified and ordered. Subsequently, gaps were filled by GapFiller (Nadalin et al., 2012) with three rounds of iterations. Finished chloroplast assembly was re-aligned to *B. hygrometrica* to confirm the quadripartite structure representing – LSC, SSC, and two IRs.

### Genome Annotation of the *Haberlea rhodopensis* Chloroplast Genome and Comparative Plastomics

The assembled chloroplast sequence was annotated using Dual Organellar Genome Annotator (DOGMA) (Wyman et al., 2004) with manual start and stop codon validation by using the Sequin tool from NCBI^2^. The nomenclature of cp genes was used according to Chloroplast Genome Database^3^ and previously published cp genomes. Transfer RNA (tRNA) genes were identified with DOGMA and the tRNAscan-SE program ver. 1.21 (Schattner et al., 2005). Circular representation of the *H. rhodopensis* chloroplast genome was generated with OGDRAW tool (Lohse et al., 2013). The complete cp genome of *H. rhodopensis* was compared with the cp genomes of *B. hygrometrica, Lavandula angustifolia, Rosmarinus officinalis, Sesamum indicum, Salvia miltiorrhiza*, and *Olea europaea* using mVista in Shuffle-LAGAN mode and Blast Ring Image Generator (BRIG). *H. rhodopensis* was set as a reference. The comparison of IR/LSC and IR/SSC regions was conducted using the GenBank genome files for *Boea hygrometrica* (NC_016468.1), *Olea europaea* (NC_013707.2) and *Sesamum indicum* (NC_016433.2) with coordinates for the gene features. IR/SSC border coordinates were obtained from previous studies (Mariotti et al., 2010; Zhang et al., 2012, 2013). For the identification of perfect and compound simple sequence repeats (SSRs), MISA^4^ was used with a minimum repetitive stretch of 10 nucleotides as mono-, a consecutive stretch of four repeats units to be classified as di- and tri-, and a stretch of three repeat units for each tetra-, penta-, and hexa nucleotide stretches as SSRs. For the identification of the dispersed repeats including the forward and palindromic repeats, REPUTER (Kurtz et al., 2001) was used with parameters (repfind -f -p -l 30 -h 3 -best 10000) and the identified repeat regions are checked with the corresponding genomic coordinates and were annotated.

### Molecular Evolution Analysis

For the identification of codon usage patterns, all coding sequences (CDSs) shorter than 300 bp were removed. Filtered CDSs were subsequently used for the estimation of codon usage using CodonW^5^ with translational table = 11. In addition to the overall codon usage, we further tabulated additional codon usage measures such as Nc (effective number of codons), GC_3s_ (frequency of the GC at the third synonymous position) (Hu et al., 2015). All the calculations of the GC at the first, second, and third position as defined by GC, GC_1_, GC_2_, and GC_3,_ respectively, was done using in-house PERL scripts. Estimation of the standard effective number of codon (Nc) was tabulated using the equation N(c) = 2 + s + 29/[s(2) + (1 – s)(2)], where s denotes GC3s (Wright, 1990). Ka/Ks value for each gene was calculated using the KaKs_calculator (Wang et al., 2010) with the following settings: genetic code table 11 (bacterial and plant plastid code); method of calculation: YN. In the results, the indication for Ka/Ks “NA” which appears when Ks = 0 (in cases with no substitutions in the alignment, or 100% match) was replaced in all cases with 0. Taking into account that KaKs Calculator estimates selection using model averaging, we also evaluated the role of the site-specific selection in 34 genes present across the 33 phylogenetically related species (**Supplementary Table S1**). For the identification of the site-specific selection, trimmed codon alignments were analyzed using Selecton (Stern et al., 2007), taking into account two models: M8 (model of positive selection) and M8a (null model) and likelihood scores estimated by models were evaluated using log-likelihood ratio test (LRT) with degree of freedom = 1. *H. rhodopensis* was used as a reference sequence to estimate the site-specific selection models in Selecton. We considered that genes with a *p*-value for the LRT below 5% (i.e., *p*-value < 0.05) to exhibit signatures of sites under positive selection.

### Phylogenetic Profiling

For understanding evolution of the coding regions, codon alignment of the coding regions of *H. rhodopensis* and 32 other chloroplast genomes (**Supplementary Table S1**) for genes *atpA. atpB. atpE. atpF. atpH. atpI, ndhC. ndhD. ndhE. ndhF. ndhH. ndhI. ndhJ, petA, petB, petD, petG, psaA, psaB, psaC, psaI, psbA, psbB, psbC, psbD, psbE, psbH, psbK, psbL, psbM, psbN, rbcL, rpoA, rpoB, rpoC2* was done using MACSE, which allows the identification of frameshift events (Ranwez et al., 2011). Following codons alignments, alignments were trimmed for the ambiguity and the concatenated alignment was analyzed for phylogenetic reconstruction using the IQTree by Maximum Likelihood method (Nguyen et al., 2015) with 1000 bootstrap replicates. Model selection for the phylogenetic assessment was done based on the Akaike Information Content (AIC) and corrected AIC. Bootstrap values were calculated using the in-built UFBoot within IQTree, and rapid bootstrap calculation and avoid less biased support values estimation (Minh et al., 2013).

### RNA Editing

Prediction of the RNA editing events was done using the BLASTX prediction mode of PREPACT2^6^ and all the 17 chloroplast reference genomes were selected to predict the RNA editing events. For the prediction of the RNA Editing events, we have kept only those sites which have 100% prediction probability and are represented across all reference genomes included in PREPACT 2.0. Black font indicates a pre-edited state and red font indicates an editing event to reconstitute a conserved codon in a given reference, respectively.

### Data Deposition

The complete chloroplast genome of *Haberlea rhodopensis* has been submitted to GenBank with accession number KX657870. The raw Illumina reads were submitted to NCBI SRA archive in FASTQ format with accession numbers SRR4428742, SRR4428743, PRJNA348808.

## Results and Discussion

### *Haberlea rhodopensis* Chloroplast Genome Organization and Gene Content

**FIGURE 1:**
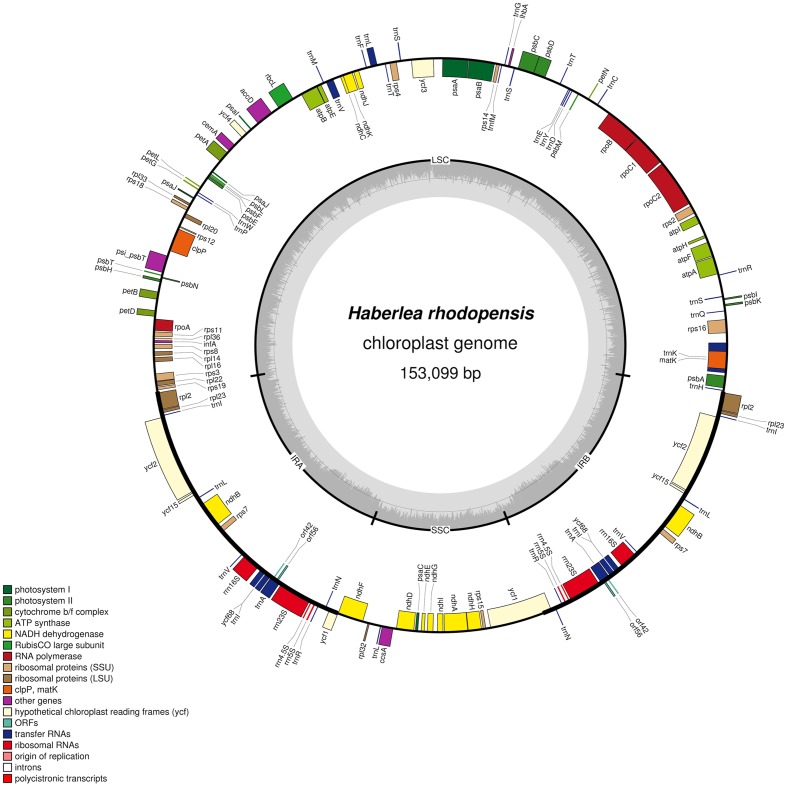
**Circular map of the chloroplast genome of *H. rhodopensis*: Genes drawn within the circle are transcribed clockwise, while genes drawn outside are transcribed counterclockwise.** Genes belonging to different functional groups are color coded. Dark bold lines show Inverted repeats (IRa, IRb). The dashed area in the inner circle indicates CG content in the chloroplast genome. The map is drawn by OGDRAW.

The complete cp genome sequence of *H. rhodopensis* (submitted to GenBank under Acc. No KX657870) is 153,099 bp in length and has the common quadripartite structure found in most land plants. The genome includes a LSC of 84,443 bp (covering 55.2%) and a SSC of 17,826 bp (covering 11.6%), separated by two IRs of 25,415 bp (covering 33.20 %) (**Figure 1**). Overall, the *H. rhodopensis* cp genome CG content was 37.8%, with a higher GC content in the IRs regions (43.3%) than in LSC and SSC (35.7 and 31.7%, respectively). Most of the cp genome sequence encodes proteins, tRNAs and rRNAs (51.56, 1.78, and 5.90%, respectively). The remaining regions are non-CDSs, including introns, intergenic spacers and pseudogenes. There are in total 137 genes in the genome, including 86 protein-coding genes, 36 tRNAs genes, 4 rRNAs, 4 pseudogenes, and 21 duplicated genes (**Table 1**). Of the observed gene space in *H. rhodopensis*, 80 protein-coding genes, 29 tRNA, and 4 rRNAs were found to be unique while six protein-coding (*rpl2, rpl23, ycf2, ycf15, ndhB, rps7*), seven tRNAs (*trnI-CAU,trnL-CAA, trnV-GAC,trnI-GAU, trnA-UGC,trnR-ACG, trnN-GUU*), and four rRNA genes (*rrn4.5, rrn5, rrn16, rrn23*) were found be duplicated in IRa and IRb (**Table 1**). Moreover, we identified four pseudogenes (*ycf1, ycf68, orf42, orf56*). Among the genes that underwent pseudogenization, only *ycf1* showed incomplete duplication (in the IRa and SSC junction region), the others – *ycf68, orf42*, and *orf56* showed complete duplication in IRa/IRb suggesting a loss of function due to the accumulation of premature stop codons or truncations. The cp genome has 13 intron-containing genes, of which eight protein-coding genes (*rps16, atpF, rpoC1, ycf3, clpP, rpl2, ndhB. ndhA*) and five tRNA genes (*trnK-UUU, trnL-UAA, trnV-UAC, trnI-GAU. trnA-UGC*). Two of the intron-containing genes have two introns (*clpP, ycf3*) and the other 15 have only one intron (**Table 2**). In addition, four cases of overlapping genes were observed (*psbD-psbC, ndhK-ndhC, atpE-atpB, ycf1-ndhF*).

**Table 1:** List of genes encoded by *Haberlea rhodopensis* chloroplast genome.

Category	Gene group	Gene name				
**Genes for photosynthesis**	Subunits of photosystem I	*psaA*	*psaB*	*psaC*	*psaI*	*psaJ*
	Subunits of photosystem II	*psbA*	*psbC*	*psbD*	*psbE*	*psbF*
		*psbH*	*psbI*	*psbJ*	*psbK*	*psbL*
		*psbM*	*psbN*	*psbT*	*psi_psbT*	
	Subunits of cytochrome b/f complex	*petA*	*petB*	*petD*	*petG*	*petL*
		*petN*				
	Subunits of ATP synthase	*atpA*	*atpB*	*atpE*	*atpF^b^*	*atpH*
		*atpI*				
	Large subunit of RuBisCo	*rbcL*				
	Subunits of NADH dehydrogenase	*ndhA^b^*	*ndhB^a,b^*	*ndhC*	*ndhD*	*ndhE*
		*ndhF*	*ndhG*	*ndhH*	*ndhI*	*ndhJ*
		*ndhK*				
**Self-replication**	Ribosomal RNA genes	*rrn4.5^a^*	*rrn5^a^*	*rrn16^a^*	*rrn23^a^*	
	Transfer RNA genes	*trnA-UGC^a,b^*	*trnC-GCA*	*trnD-GUC*	*trnE-UUC*	*trnF-GAA*
		*trnG-UCC*	*trnH-GUG*	*trnI-CAU^a^*	*trnI-GAU^a,b^*	*trnK-UUU^b^*
		*trnL-CAA^a^*	*trnL-UAA^b^*	*trnL-UAG*	*trnM-CAU*	*trnfM-CAU*
		*trnN-GUU^a^*	*trnP-UGG*	*trnQ-UUG*	*trnR-ACG^a^*	*trnR-UCU*
		*trnS-GCU*	*trnS-GGA*	*trnS-UGA*	*trnT-GGU*	*trnT-UGU*
		*trnV-GAC^a^*	*trnV-UAC^b^*	*trnW-CCA*	*trnY-GUA*	
	Ribosomal proteins (SSU)	*rps2*	*rps3*	*rps4*	*rps7^a^*	*rps8*
		*rps11*	*rps12*	*rps14*	*rps15*	*rps16^b^*
		*rps18*	*rps19*			
	Ribosomal proteins (LSU)	*rpl2^a,b^*	*rpl14*	*rpl16*	*rpl20*	*rpl22*
		*rpl23^a^*	*rpl32*	*rpl33*	*rpl36*	
	RNA polymerase	*rpoA*	*rpoB*	*rpoC1^b^*	*rpoC2*	
	Translational initiation factor	*infA*				
**Other genes**	Maturase	*matK*				
	Envelope membrane protein	*cemA*				
	LHC of PSII associated factor 1	*lhbA*				
	Subunit of acetyl-CoA	*accD*				
	C-Type cytochrome synthesis gene	*ccsA*				
	Protease	*clpP^c^*				
	Hypothetical chloroplast reading	*ycf1^a,d^*	*ycf2^a^*	*ycf3^c^*	*ycf4*	*ycf15^a^*
	Frames	*ycf68^a,d^*				
	ORF	*orf56^a,d^*	*orf42^a,d^*			


*(a) Two gene copies in IRs; (b) gene containing a single intron; (c) gene containing two introns; (d) pseudogene.*

**Table 2:** Location and length of intron-containing genes in *Haberlea rhodopensis* chloroplast genome.

Gene	Location	Exon I (bp)	Intron I (bp)	Exon II (bp)	intron II (bp)	Exon II (bp)
*rps16*	LSC	48	914	213		
*atpF*	LSC	144	656	471		
*rpoC1*	LSC	456	781	1620		
*ycf3*	LSC	129	703	228	714	153
*clpP*	LSC	69	808	291	615	228
*rpl2^∗^*	IR	393	669	435		
*ndhB^∗^*	IR	777	680	756		
*ndhA*	SSC	558	941	540		
*trnK-UUU*	LSC	37	2527	26		
*trnL-UAA*	LSC	37	475	50		
*trnV-UAC*	LSC	38	582	37		
*trnI-GAU^∗^*	IR	42	936	35		
*trnA-UGC^∗^*	IR	38	823	35		


*^∗^Identical duplicate gene containing introns in the IR region are not included.*

Plant cells often contain multiple copies of chloroplast genomes (Krebs et al., 1987) which can be regarded as genetic heterogeneity within a population (Yang et al., 2010). We mapped all Illumina reads to the assembled genome to identify the possible polymorphic sites. However, no polymorphisms were recovered, which was also observed in a cp genome from the family Gesneriaceae (*B. hygrometrica*, Zhang et al., 2012).

### Comparative Analysis of the *Haberlea rhodopensis* Chloroplast Genome

**FIGURE 2:**
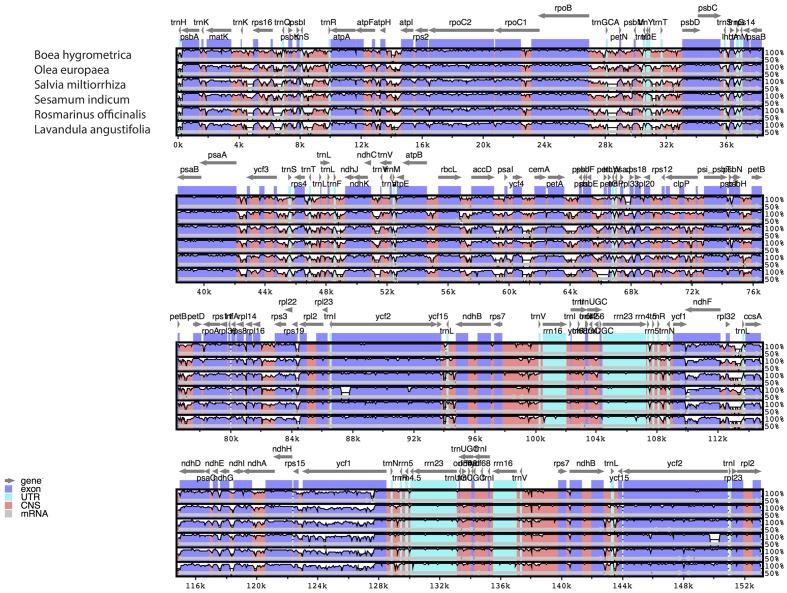
**Sequence identity plots (mVISTA) among six species of Lamiales, using *H. rhodopensis* as reference genome.** Arrows indicate the annotated genes and their transcriptional direction. Genome regions are color coded as exon, untranslated region (UTR), conserved non-coding sequences (CNS), and mRNA.

The comparative analysis between the cp genomes of *H. rhodopensis* and *B. hygrometrica* is shown in **Table 3**. As expected, conserved synteny and gene order conservation was observed, which might be due to the family level conservation as both species belong to the family Gesneriaceae. Furthermore, a multiple sequence alignment (MSA) based on mVISTA (Poliakov et al., 2014) and BRIG (Alikhan et al., 2011) (**Supplementary Figure S1**) was performed among six closely related cp genomes for investigating levels of sequence divergence between them (**Figure 2**). Pairwise chloroplast genomic alignment between *H. rhodopensis* and other genomes also revealed a high degree of synteny suggesting the evolutionary conservation of these genomes at the genome-scale level. The complete aligned sequences revealed that Lamiales cp genomes possess high sequence similarity which suggests the genomes are rather conservative, although some divergent regions were found as well. As seen in other flowering plants (Nie et al., 2012; Dong et al., 2013; Liu et al., 2013), coding regions were more conserved than their non-coding counterparts. The most dissimilar coding regions among aligned cp genomes were *ycf1, ndhF, accD, ccsA, rbcL, ycf2*, and *rps19*. The *ycf1, rbcL*, and *accD* coding regions have also been observed as divergent in plastomes of other angiosperms (Nie et al., 2012; Dong et al., 2013, 2015; Liu et al., 2013; Luo et al., 2014) that makes such genes reliable markers for phylogenetic analysis (Nazareno et al., 2015). In contrast to *S. indicum* and *S. miltiorrhiza*, where *rps19* is duplicated, *B. hygrometrica* is the only species in Gesneriaceae that has been reported to contain pseudogene *rps19* (Zhang et al., 2012), but is was observed as protein coding gene in *H. rhodopensis*. The IR regions are less divergent than SSC and LSC regions. The non-coding regions showing higher sequence divergence were *petA-psbJ, psbE-petL, ycf4-cemA, atpH-atpI, ndhC-trnV*. Due to this fact, some of these chloroplast non-coding regions have been used in phylogenetic studies (Shaw et al., 2007; Wu et al., 2010; Nie et al., 2012). Another interesting observation from the comparative point of view (with the closest member of the same family) is that the genes *psbI* and *rps12* are found in *B. hygrometrica* as pseudogenes, whereas in *H. rhodopensis* we found them to be normal protein-coding genes.

**Table 3:** Comparison of genome contents of *H. rhodopensis* and *B. hygrometrica.*

	*H. rhodopensis*	*B. hygrometrica*
Total sequence length (bp)	153, 099	153, 493
Large single copy (bp)	84, 443	84, 692
Small single copy (bp)	17, 826	17, 901
Inverted repeat region (bp)	25, 415	25, 450
GC% content	37, 8%	37, 59%
Total CDS bases (bp)	79747	79218
Average CDS length (bp)	917	932
Total RNA bases (bp)	11769	11780
Average intergenic distance (bp)	390	396.66


The intron-containing genes in *H. rhodopensis* cp genome are summarized in **Table 2**. Unlike some very close Lamiales members (*S. indicum* and *S. miltiorrhiza*), several introns are lacking in *trnG-UCC, rps12, petB, petD, rpl16* in the plastome of *H. rhodopensis*, similar to *B. hygrometrica*. Similar structures are reported also in these genes in *Tanaecium tetragonolobum* (Nazareno et al., 2015).

**FIGURE 3:**
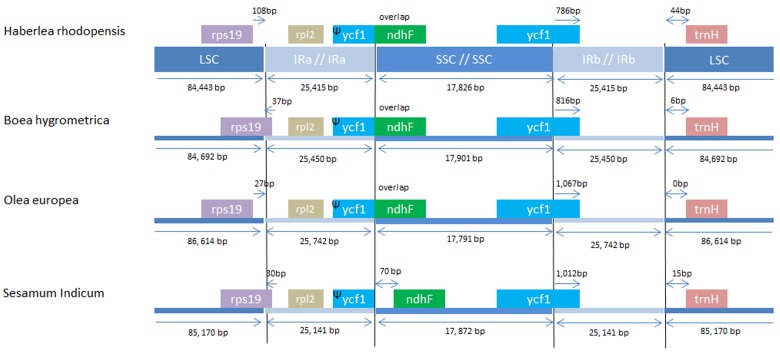
**Comparison of chloroplast borders of LSC, SSC, and IRs among the species from Lamiales**.

The IRs regions are one of the most conserved regions in the cp genomes among all species. The IR boundary contraction and expansion are regarded as evolutionary events and are showed to be the main reason for size variation in cp genomes. These junctions are regarded as an index of chloroplast genome evolution (Zhang et al., 2013). The IR/LSC and IR/SSC regions of *H. rhodopensis* cp genome were compared to the corresponding regions of the closely related cp genomes of *B. hygrometrica, O. europaea, S. indicum* (**Figure 3**). As observed in other chloroplast genome studies (Nazareno et al., 2015), the IR expansion/contraction in Lamiales has led to changes in the structure of the chloroplast genome, contributing to the formation of pseudogenes. The IRa/SSC border extended into *ycf1* resulting in à pseudogene in the four compared cp genomes. The length of *ycf1* pseudogene was 775 bp in *H. rhodopensis*, 1,115 bp in *O. europaea*, 813 bp in *B. hygrometrica* and 1,012 bp in *S. indicum*. Moreover, there is overlapping of *ycf1* pseudogene and *ndhF* in *H. rhodopensis. B. hygrometrica, O. europaea*, and *S. indicum*. The IRb/SSC region was located in the CDS of *ycf1* gene in all compared cp genomes. The *trnH* genes of compared species were located in LSC region, 0–44 bp apart from the IRb/LSC border. Interestingly, the *rps19* gene was situated in LSC region of *H. rhodopensis* and *O. europaea* genomes, separated from the IRa/LSC region by 27 and 108 bp, respectively, whereas it was extended into IRa in *B. hygrometrica* and *S. miltiorrhiza* and *S. indicum*.

### Repeat Analysis

Organelle genomes, especially chloroplast, have long been used as a source to understand the phylogenetic relationship of species, mainly due to its uniparental inheritance mode and also due to its rapidly evolving gene content (Sablok et al., 2013). Taking into account the role of the organelle SSRs as important phylogenetic markers, we analyzed the distribution of SSRs with following length thresholds of minimum repetitive units: 10 repeats for mono-, 4 repeats for di- and tri-, and 3 repeats for tetra-, penta-, and hexa-nucleotide repeat patterns. We observed a total of 71 SSRs patterns with 10 SSRs present in compound patterns (**Table 4**). Among the identified repeat patterns, dinucleotide repeat patterns (AG/CT and AT/TA) formed the most abundant repeat patterns. Our finding agrees with the observation that cp SSRs are generally composed of short polyadenine (polyA) or polythymine (polyT) repeats and rarely contain tandem guanine (G) or cytosine (C) repeats (Kuang et al., 2011). In total, 29 SSRs were found to be present in the genic regions in *H. rhodopensis*. Among them, seven genes (*atpF, rpoC2, rpoA, rpl2, ycf2, ndhB, rrn23*) were found to harbor at least two SSRs. It is interesting to see that the number of identified SSRs is low compared to the previously characterized SSRs in organelle genomes (e.g., *B. hygrometrica, O. europaea, and S. indicum*, Mariotti et al., 2010; Yi and Kim, 2012; Zhang et al., 2012). Additionally, we did not find a large abundance of tri- to tetra-nucleotide repeats, which is in contrast to previous reports (Qian et al., 2013). We did not observe any penta- or hexa-nucleotide repeat patterns in *H. rhodopensis*. This is similar to the chloroplast genome of *Utricularia reniformis* belonging to Lamiales, which lacks penta- or hexa-nucleotide repeats (Silva et al., 2016). The identified SSRs, together with the provided primer pairs, could be used for determination of the phylogeography and population structure pattern of *H. rhodopensis* (**Supplementary Table S3**).

**Table 4:** Cumulative SSR frequency and corresponding primer pairs in *Haberlea rhodopensis* (SSR search parameters: 1–10; 2–4; 3–4; 4–3; 5–3; 6–3 where 1, 2, 3, 4, 5, and 6 indicate the mono- di-, tri-, tetra-, penta-, and hexa-nucleotide repeats).

Total size of examined sequences (bp):	153099
Total number of identified SSRs:	71
Number of SSRs present in compound formation:	10

**SSR type**	**Frequency**
A/T	24
C/G	1
AC/GT	4
AG/CT	15
AT/AT	19
AAG/CTT	1
AAT/ATT	1
AAAC/GTTT	1
AAAT/ATTT	2
AACT/AGTT	1
AATC/ATTG	1
AGAT/ATCT	1


*SSR type – type of detected SSRs in *H. rhodopensis* genome.*

In addition to the SSRs, we further explored the role of the long repeats as identified by REPUTER (Kurtz et al., 2001). We identified a total of 40 repeats – 18 forward and 22 palindromic (inverted) repeats (**Table 5**). These repeats were found to be at least 30 bp per unit and the biggest was 59 bp. Around 40% of these repeats fell exclusively into intergenic regions, whereas 60% are situated in genes or at their border. In contrast to some closely related species such as *S. indicum* with 15 repeats, *B. hygrometrica* with eight repeats, and *O. europaea* with three repeats (Nazareno et al., 2015), *H. rhodopensis* contains a higher number of repeat elements. Most of these repeats exhibit lengths between 30 and 44 bp, while the *ycf2* CDS possesses the highest number of repeats (11) and *ycf1* has the longest repeats at 59 bp. The localization of forward repeats can be a consequence of the plastomic rearrangements and can provide important clues toward understanding the spatial-temporal organization in Geraniaceae (Huang et al., 2013).

**Table 5:** Distribution and localization of repeat sequences in cpDNA of *Haberlea rhodopensis.*

Size (bp)	Start position1	Start position2	Type	Location	Region	*E*-value
59	109798	109857	F	ycf1	IRA	1.98e-26
59	109857	127696	P	ycf1	SSC	1.98e-26
56	84299	84443	F	rps19, IGS	LSC	1.27e-24
56	84299	153043	P	rps19, IGS(rpl2,trnH-GUG)	IRA, IRB	1.27e-24
55	36105	36159	F	IGS(trnS-UGA,lhbA)	LSC	5.08e-24
41	98642	119664	F	IGS(rps7,trnV-GAC),ndhA	IRA, SSC	1.36e-15
41	119664	138870	P	ndhA, IGS(trnV-GAC,ndhB)	SSC, IRB	1.36e-15
40	59562	59562	P	IGS(accD, psaI)	LSC	5.45e-15
37	57457	57494	F	IGS(rbsL, accD)	LSC	3.49e-13
44	38927	41151	F	psaB, psaA	LSC	7.62e-12
37	6418	80983	F	IGS(rps16,trnQ-UUG), IGS(rps8,rpl14)	LSC	3.87e-11
33	113317	113349	F	IGS(rpl32,trnL-UAG)	SSC	8.93e-11
42	43919	119663	F	ycf3,ndhA	LSC, SSC	1.06e-10
39	43922	138870	P	ycf3, IGS(trnV-GAC,rps7)	LSC, IRB	1.45e-10
39	43922	98644	F	ycf3, IGS(rps7, trnV-GAC)	LSC, IRA	1.45e-10
31	162	199	P	IGS(trnH-GUG,psbA)	LSC	1.43e-09
30	8063	45627	P	IGS(psbI, trnS-GCU)	LSC	5.72e-09
33	63845	63845	P	IGS(petA, psbJ)	LSC	8.84e-09
36	91664	145820	P	ycf2	IRA, IRB	2.69e-07
36	91664	91682	F	ycf2	IRA	2.69e-07
36	91682	145838	P	ycf2	IRA, IRB	2.69e-07
36	145820	145838	F	ycf2	IRB	2.69e-07
35	94093	94093	P	IGS(ycf15, trnL-CAA)	IRA	9.87e-07
35	94093	143408	F	IGS(ycf15,trnL-CAA), IGS(trnL-CAA,ycf15)	IRA, IRB	9.87e-07
35	143408	143408	P	IGS(trnL-CAA, ycf15)	IRB	9.87e-07
32	8501	8501	P	IGS(trnS-GCU,trnR-UCU)	LSC	1.60e-06
34	74589	74599	P	IGS(psbT, psbN)	LSC	3.61e-06
31	20927	20929	P	rpoC1	LSC	5.98e-06
30	13264	13264	P	IGS(atpF, atpH)	LSC	2.24e-05
31	54809	66123	P	IGS(atpB,rbcL), IGS(psbE,petL)	LSC	1.73e-04
30	9660	36718	F	IGS(trnS-GCU,trnR-UCU), trnG-UCC	LSC	6.27e-04
30	43934	138867	P	ycf3, IGS(trnV-GAC, rps7)	LSC, IRB	6.27e-04
30	43934	98656	F	ycf3, IGS(rps7, trnV-GAC)	LSC, IRA	6.27e-04
30	89251	148215	P	ycf2	IRA, IRB	6.27e-04
30	89251	89293	F	ycf2	IRA	6.27e-04
30	89293	148257	P	ycf2	IRA, IRB	6.27e-04
30	91675	145815	P	ycf2	IRA, IRB	6.27e-04
30	91675	91693	F	ycf2	IRA	6.27e-04
30	91693	145833	P	ycf2	IRA, IRB	6.27e-04
30	148215	148257	F	ycf2	IRB	6.27e-04


*F, forward repeat; P, palindrome repeat; IGS, intergenic spacers.*

### Codon Usage and Selection Events in Protein-Coding Genes

Codon usage plays an important part in shaping the plastome evolution. Among the several features that shape codon usage, mutational bias has an essential role in shaping this evolutionary phenomenon (Liu and Xue, 2005). Several features have been shown to affect the codon usage at the mutational and translational levels, however, mutational pressure is the dominant force acting at the level of chloroplast genomes. The strand asymmetry, causing strand-specific bias in organelle genomes, is among the other factors that could contribute to shaping the codon usage bias (Jia and Higgs, 2008). For the estimation of the codon usage, a total of 58 genes were selected based on 300 bp length threshold from *H. rhodopensis* and *B. hygrometrica*. Codon usage measures such as Nc, frequency of A, T, G, and C at the third synonymous sites, aromaticity and gravy were estimated (**Supplementary Table S2**). For the estimation of the mutational bias, we evaluated mutational pressure in *H. rhodopensis* using Nc plots (**Supplementary Figure S2**). We observed that most of the genes, with a size threshold of 300 bp, fall below the expected line of Nc thus suggesting that mutational bias is a dominant factor shaping the codon usage patterns in *H. rhodopensis* (**Supplementary Table S2**). Furthermore, we evaluated the spearman rank correlation between the Nc and GC_3s_ (*R* = 0.544; *p* > 0.0001), which further indicates the role of the mutational pressure in *H. rhodopensis*. We further evaluated the role of mutational bias in phylogenetically close *B. hygrometrica* and also observed a strong positive correlation between Nc and GC_3s_ (*R* = 0.4542; *p* > 0.0001), in agreement with an important role of mutational bias in members of the Gesneriaceae family.

The synonymous and non-synonymous nucleotide substitution patterns are very important markers in gene evolution studies. In most genes except for the very rapidly evolving ones, non-synonymous nucleotide substitutions have occurred less frequently than synonymous substitutions due to the action of purifying selection. Accordingly, the ratio of Ka/Ks < 1 (especially less than 0.5) indicates purifying selection; Ka/Ks > 1 indicates probable positive selection whereas Ka/Ks values close to 1 indicate neutral evolution, or relaxed selection (Kimura, 1983). We calculated Ka/Ks ratios of *H. rhodopensis* chloroplast genome versus three closely related Lamiales species: *B. hygrometrica, S. indicum, S. miltiorrhiza* and the more distantly related *Coffea arabica* (**Supplementary Table S4**). Our analysis showed that the Ka/Ks ratios are not region-specific (the IR, SSC, and LSC regions showed comparable values) but are mostly gene-specific. The average Ka/Ks ratio for 72 protein genes analyzed in the five genomes was 0.2139. The most conserved genes with average Ka/Ks values between 0 and 0.01, indicating very strong purifying selection pressure are *atpH, psaA, psaB, psaC; psbA, psbC, psbD, psbE, psbF, psbH, psbL, psbM, psbN, psbT, petG, petL*, and *infA*. Model averaging method in KaKs calculator showed average Ka/Ks > 1 for *rps12* (encoding the large subunit ribosomal protein 23) and *rpl23* (encoding the ribosomal protein S12). The increased Ka/Ks ratios indicative for positive selection could reflect selection pressures specific for *H. rhodopensis* cp genome. Alternatively, they could be a sign of increased variability of the particular proteins within a broader group of species. In the first case, there could be a relationship between these selection indexes and the resurrection phenotype. To discern which is the case, we performed a cross analysis calculating Ka/Ks ratios between all other possible sequence pairs of sequences compared (*C. arabica* and *S. indicum; C. arabica* and *S. miltiorrhiza; C. arabica* and *B. hygrometrica; S. indicum* and *S. miltiorrhiza; S. indicum* and *B. hygrometrica; S. miltiorrhiza* and *B. hygrometrica*) for the genes *rps12 and rpl23.*

The Ka/Ks cross test showed that the positive selection events for these genes were specific for *H. rhodopensis. rpl23* is one of the candidates for adaptive evolution. The *rpl23* average Ka/Ks value between *H. rhodopensis* and the other four species was 2.154, whereas the average Ka/Ks value for the rest of the analyzed pairs was 0.298 (**Supplementary Table S4**). *rps12* is another gene with indications for positive selection. This gene showed a very high average Ka/Ks ratio – 2.15 for the pairs of *H. rhodopensis* vs. the other four species, in comparison to 0.633 for the pairs not including *H. rhodopensis*.

Both *rpl23* and *rps12* have been proven to be essential for the chloroplast ribosome (Fleischmann et al., 2011; Tiller and Bock, 2014). The bacterial ortholog of *rpl23* in *E. coli* is located next to the tunnel exit of the large subunit, where it participates in the formation of a cradle-like surface embracing the polypeptide exit region. The ribosome structure and topology are highly conserved between bacteria and chloroplasts. As it was revealed in a three-dimensional cryo-EM map of the spinach 70S chloro-ribosome, the chloroplast *rpl23* protein is located at the same position on the chloro-ribosome as its bacterial ortholog and probably performs a similar function (Sharma et al., 2007). The positive selection pressure on *rpl23* sequence in *H. rhodopensis* genome could reflect rapid adaptive changes in the machinery during the biosis–anabiosis transitions of this resurrection species. Interestingly, we did not observe signs of positive selection for this gene in the closely related genome of *Boea hygrometrica* (another resurrection species) so this is most probably a recent adaptation unique for *Haberlea rhodopensis*.

A similar rationale could be applied to the *rps12* gene, which codes for an important protein of the small ribosome subunit. Studies in Chlamydomonas reveal that Rps12 protein plays an important role in the decoding center of chloroplast ribosomes and its absence leads to a general block of chloroplast translation. Repression of the *rps12* gene leads to the arrest of cell growth and induces a response that involves expression changes in nuclear-encoded genes for plastid biogenesis, protein turnover, and stress. This response also leads to the overaccumulation of several plastid transcripts. It is an indication of the existence of complex negative regulatory feedback loops in the chloroplast gene circuitry (Ramundo et al., 2013). The chloroplast ribosomes and translational apparatus are important participants in the retrograde signaling and play a key role in the regulation of important signaling cascades related to plant growth and stress responses (Fleischmann et al., 2011). In this respect, the positive selection signatures on the sequences of the two ribosomal protein genes in *Haberlea* genome could be regarded and further analyzed in a broader perspective.

**FIGURE 4:**
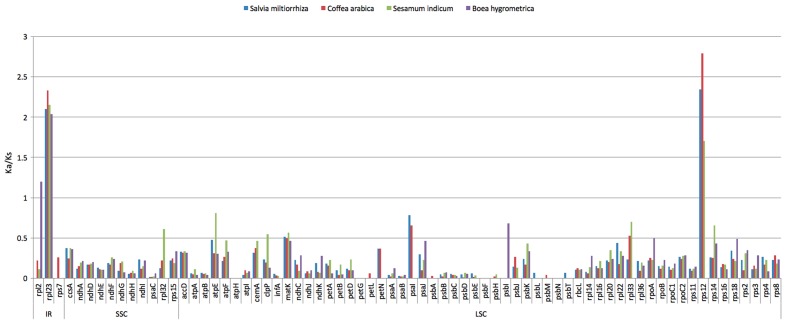
**The Ka/Ks ratio of 72 protein-coding genes of four cp genomes for comparison with *H. rhodopensis***.

Values of Ka/Ks in the range of 0.5 to 1.0 (indicating relaxed selection) were observed for the genes *rpl2, rpl32, atpE, psaI, psbI, matK* and *clpP* (**Figure 4**). The Ka/Ks values of the rest of the genes were between 0.02 and 0.49, which means most of the genes in the cp genome of *H. rhodopensis* are under purifying selection.

*H. rhodopensis* is interesting with its homoiochlorophyllous resurrection phenotype, and with the rapid recovery of its photosynthetic levels upon rehydration. The possible relaxed selection pressure on psaI and psbI, which encode integral thylakoid membrane proteins, may reflect recent adaptations of the photosynthetic apparatus of this species to repeated desiccation and rehydration. The relaxed selection for the ATP synthase subunit *atpE*, along with the presence of positively selected sites in its sequence (see below) could also be related to adaptations to desiccation stress.

In addition to the Ka/Ks analysis, we also identified the site-specific selection events using the Selecton (Stern et al., 2007). Selecton analysis revealed a total of 17 genes displaying site-specific selection (**Table 6**). Interestingly, *rbcL* was found to harbor 13 sites under positive selection. This gene has been previously used to establish the diverse phylogenetic relationships in the resurrections plants in Selaginellaceae (Korall and Kenrick, 2002). *rbcL* plays an important role as a modulator of photosynthetic electron transport and is essential for photosynthesis (Allahverdiyeva et al., 2005). Previous estimates of the desiccation in resurrection plants indicate a physiological basis of the slow recovery of the assimilatory action of the photosynthetic carbon (Schwab et al., 1989). Identification of the positive sites in this study could lead to the establishment of the photosynthetic mutants that could lead to the physiological understanding of the desiccation in resurrection plants. In addition, to *rbcL*, we also observed site-specific selection in *atpE*, which is a co-transcriptionally coupled gene with *atpB* (Chotewutmontri and Barkan, 2016) and plays a critical role in the activation and de-activation of the CF_0_CF_1_, which is a H^+^ translocating ATPase. A key feature of the homoiochlorophyllous resurrection plants is their ability to maintain the integrity of the photosynthetic apparatus and its re-activation following the rehydration. High rates of evolvability in *atpE* might indicate the role of selection in fine-tuning the demands of the rapid activation of the ATP synthase and to increase the co-transcription of the *atpE* with *atpB* to meet the metabolic demand for ATP and NADPH in resurrection plants (Rott et al., 2011). Furthermore, we also detected site-specific selection events in *accD*, which have been shown to affect the plant fitness by altering the acetyl-CoA carboxylase production (Madoka et al., 2002). In addition to these genes, we observed site-specific selection in *ndhF*, which is critical to the onset and delay in senescence. The observed site-specific selection events in the *ndhF* gene might affect the translational output of *ndhF* gene and thus may play a role in the stress adaptation of *H. rhodopensis* by delaying drought-induced senescence. It is worth mentioning that previously *ndhF* mutants (DeltandhF) with the plastid *ndhF* gene knocked-out in transgenic tobacco showed 30-day-delayed senescence (Zapata et al., 2005).

**Table 6:** Positive selection sites identified with selection with *df* = 1.

Gene	NULL (M8a)	POSITIVE (M8)	Putative sites under positive selection
*atpE*	–2156.21	–2153.31	1 (131 M)
*matK*	–11147.00	–11140.60	1 (70 S)
*ndhF*	–14940.40	–14937.90	7 (468 N, 486 R, 557 L, 597 K, 604 R, 693 Y, 695N)
*ndhJ*	–2045.17	–2043.14	1 (93 S)
*psaA*	–7646.86	–7644.55	2 (165 V, 600 A)
*psaI*	–446.59	–443.27	1 (2 S)
*psbH*	–978.46	–973.65	1 (21 A)
*psbK*	–687.88	–683.14	1 (40 L)
*psbL*	–285.42	–282.01	1 (1 M)
*rbcL*	–6375.16	–6358.92	13 (86 H, 142 P, 145 T, 225 I, 251 I, 279 S, 354 V, 429 Q, 439 V,449 C, 468 N, 470 P, 472 I)
*rpl16*	–1878.87	–1874.32	2 (6 L, 10 S)
*rpl22*	–2824.08	–2819.11	5 (73 L, 107 D, 108 K, 110 E, 113 R)
*rpoC2*	–22883.50	–22872.20	11 (522 Q, 536 Y, 545 H, 549 H, 577 Q, 725 K, 727 S, 935 S, 941 L, 1208 V, 1268 H)
*rps4*	–2736.18	–2733.16	3 (33 K, 154 Q, 164 K)
*rps8*	–2276.40	–2273.57	2 (57 Y, 72 I)
*rps18*	–1419.21	–1417.18	3 (79 Q, 85 T, 93 N)
*accD*	–7447.39	–7432.39	8 (32 I, 52 W, 97 L, 142 C, 152 L, 156 A, 155 R, 298 R)


*Likelihood ratio test (LRT) analysis of models comparison M8 vs M8a, based on *k* = qchisq(1–*p. df* = 1), where for *p* = 0.05, *k* = 3.84. M8 – Model with positive selection M8a – Null model without positive selection.*

### Phylogenetic Analysis

**FIGURE 5:**
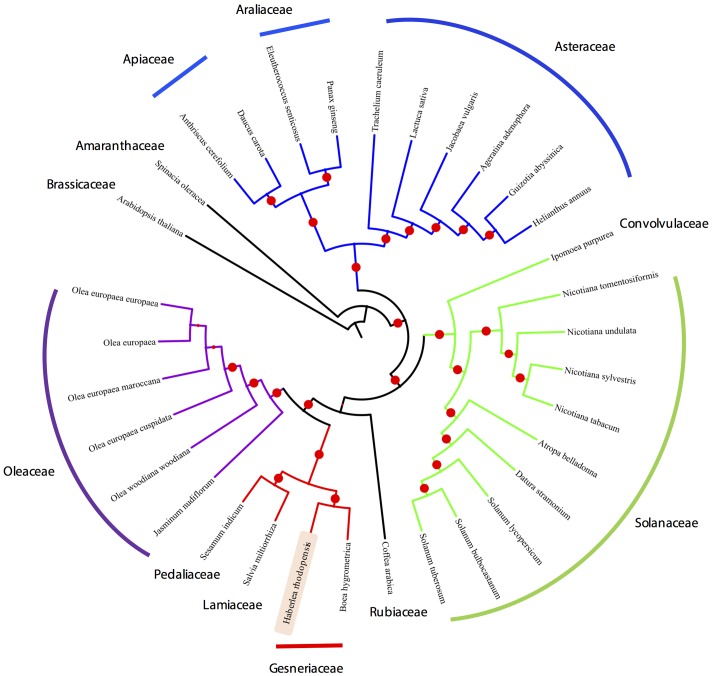
**Phylogenetic tree based on 35 protein-coding genes from 33 species and *H. rhodopensis* phylogenetic placement**.

Organelle genome sequencing plays a key role in deciphering the evolutionary phylogenomics and cladistics of plants species. For the phylogenetic reconstruction, we performed a concatenate codon-based sequence alignment of the *atpA, atpB, atpE, atpF, atpH, atpI, ndhC, ndhD, ndhE, ndhF, ndhH, ndhI, ndhJ, petA, petB, petD, petG, psaA, psaB, psaC, psaI, psbA, psbB, psbC, psbD, psbE, psbH, psbK, psbL, psbM, psbN, rbcL, rpoA, rpoB*, and *rpoC2* (Full list of species used in the phylogenetic analysis is provided in **Supplementary Table S1**). The basis of selection of these genomes for the comparative analysis is due to the fact that these genomes share the same evolutionary clade thus making them phylogenetically close. Additionally, chloroplasts from Lamiales were chosen as a closest relative outgroup to the sequenced chloroplast genome in this study, i.e., *H. rhodopensis*. For phylogenetic inferences, *Arabidopsis thaliana* has also been included as a distant outgroup. Model estimation revealed GTR+G4 on the basis of the Akaike Information Criterion (–lnL = 165400.957), Corrected Akaike Information Criterion (–lnL = 335191.641) and Bayesian Information Criterion (–lnL = 352321.906). For the maximum-likelihood analysis, 1000 bootstrap replicates were evaluated and the consensus tree from IQTree (Nguyen et al., 2015) was re-rooted using *Arabidopsis thaliana* as an outgroup, revealing the monophyletic origin of the *H. rhodopensis* which clusters with *B. hygrometrica* (**Figure 5**) (**Supplementary Data Sheet S2**). IQtree consensus tree supported the monophyletic origin of *H. rhodopensis*, which can be further used to understand the phylogenetic clade and evolution of Gesneriaceae species.

### RNA Editing Events

RNA editing is a posttranscriptional process, which mainly involves the conversion of cytidine to uridine and forms an important part of the RNA maturation process (Chen et al., 2011; Hein et al., 2016). The RNA editing factors such as CRR28 and RARE1 have been widely shown to affect the cytidine-to-uridine conversion in ndhBeU467PL, ndhDeU878SL, and accDeU794SL (Hein et al., 2016). Using PREPACT2 and parameters described as per (Hein et al., 2016), we identified a total of 17 editing sites with 100% representation across the 17 in-built organelle genomes in PREPACT2 (**Supplementary Figure S3**). In accordance with the recent studies (Hein et al., 2016), we also observed the most C-U RNA editing events in *ndh* gene, which are known to act as electro-regulators to adjust the ROS accumulation in cyclic photosynthesis electron transporters (Martín and Sabater, 2010). Although the loss of the editing events ndhBeU467PL and ndhDeU878SL has been observed in previous phylogenomic studies (Hein et al., 2016), these events were now identified in *H. rhodopensis*. In angiosperms, accDeU794SL has shown to be substantially absent in around 50% of the angiosperms. However, a loss of the accDeU794SL was not observed in the case of *H. rhodopensis*. Taking into account the conservation of the editing events, it can be presumed that resurrection plants maintain the conservatory editing events. Since *H. rhodopensis* is a desiccation-tolerant species, the identified RNA editing events could be used in future for understanding the role of the RNA editing in desiccation stress and further to understand the physiological behavior of resurrection plants.

## Conclusion

In summary, we present for the first time the complete chloroplast genome of *Haberlea rhodopensis*, a tertiary relict and Balkan endemic resurrection species from the Gesneriaceae family. The genome sequencing, assembly, annotation, and the comparative analysis, reveal that the cp genomes of *H. rhodopensis* and *B. hygrometrica* share a quadruple structure, gene order, GC content, and codon usage features, similar to those of Lamiaceae cp genomes. The phylogenetic analysis supports the monophyletic origin of *Haberlea rhodopensis* and can be used as a reliable phylogenetic framework to understand the evolution of the Gesneriaceae species. The analysis of *H. rhodopensis* cp genome uncovers several intriguing features, which can be used as a basis for the understanding the resurrection tolerance of this plant. Specifically, the *H. rhodopensis* cp genome harbors 137 genes, of which 86 are protein-coding. The results of a site-specific selection analysis point to positively selected sites in several chloroplast genes, such as *atpE, rbcL, psbI, psbA, ndhH*, and *accD*. The observed specific cp genomic features of *Haberlea rhodopensis* may be interpreted as being a consequence of molecular adaptation to drought stress, which awards an evolutionary advantage to this species.

The chloroplast genome reported in this study will make it possible to understand the function of the specific sites under selection, by developing site-directed mutagenesis assays or by point mutations at those sites. Furthermore, protein modeling will be needed in the future to analyze subtle site-specific changes resulting from recent adaptations of the photosynthetic apparatus of this species to repeated desiccation and rehydration.

## Author Contributions

ZI, GS, and VB analyzed the data; EA and GZ conducted the experimental part of the work; GY and ED contributed to the analysis; ZI, ED, and VB conceived the project and secured the funding; ZI, GS, and VB wrote the paper with contributions from all co-authors. All co-authors have read and approved the manuscript.

## Conflict of Interest Statement

The authors declare that the research was conducted in the absence of any commercial or financial relationships that could be construed as a potential conflict of interest.

## Footnotes

1 http://hannonlab.cshl.edu/fastx_toolkit/

2 http://www.ncbi.nlm.nih.gov/Sequin

3 http://chloroplast.cbio.psu.edu

4 http://pgrc.ipk-gatersleben.de/misa/

5 http://codonw.sourceforge.net/

6 http://www.prepact.de/prepact-main.php

## Supplementary Material

FIGURE S1 **Genome comparison of six chloroplast genomes to the *H. rhodopensis* chloroplast genome.** Produced alignments are color coded based on the similarity score. The first outer ring is consisting of protein-coding genes based on (corresponding to) the *H. rhodopensis* chloroplast genome.

FIGURE S2 **Estimation of the standard effective number of codon (Nc) was tabulated using the equation N(c) = 2 + s + 29/[s(2) + (1 – s)(2)], where s denotes GC3s (Wright, 1990).**

FIGURE S3 **RNA editing output from PREPACT2 tool.**

TABLE S1 **List of the chloroplast genomes used for phylogenetic analysis.**

TABLE S2 **CodonW output for genes ≥ 300 bp.**

TABLE S3 **Table showing SSRs with primer pairs.**

TABLE S4 **Ka/Ks analysis table.**

DATA SHEET S1 **Merged read quality, length distribution and QUAST results.**

DATA SHEET S2 **IQtree consensus tree.**
