# Supplementary figures and images for: Chloroplast Genome Analysis of Resurrection Tertiary Relict Haberlea rhodopensis Highlights Genes Important for Desiccation Stress Response
Source: Front Plant Sci. 2017 Feb 20;8:204. doi: 10.3389/fpls.2017.00204 (PMC5316520; doi:10.3389/fpls.2017.00204)

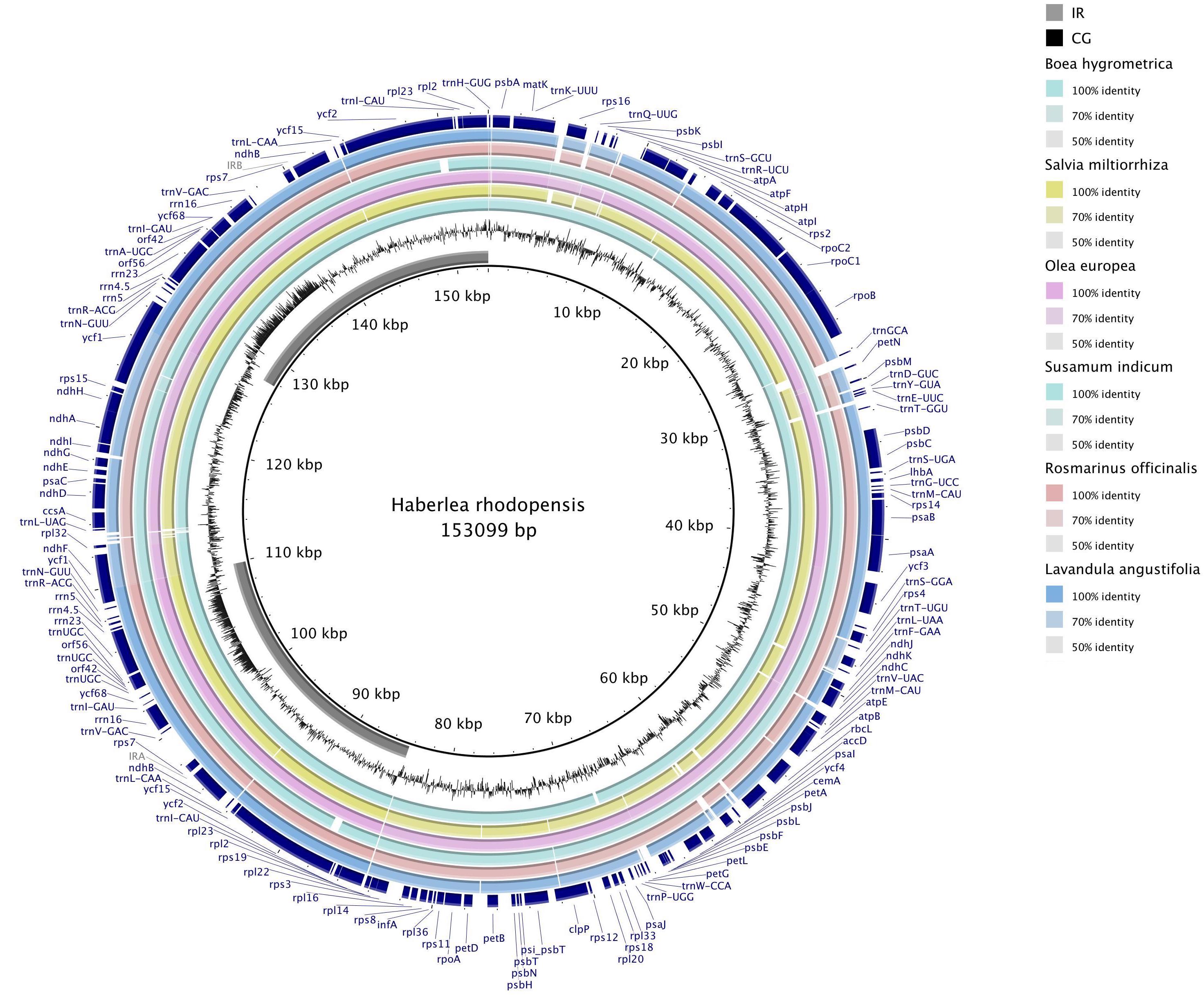

Supplement: FIGURE S1 — Genome comparison of six chloroplast genomes to the H. rhodopensis chloroplast genome. Produced alignments are color coded based on the similarity score. The first outer ring is consisting of protein-coding genes based on (corresponding to) the H. rhodopensis chloroplast genome. [file Image_1.JPEG]

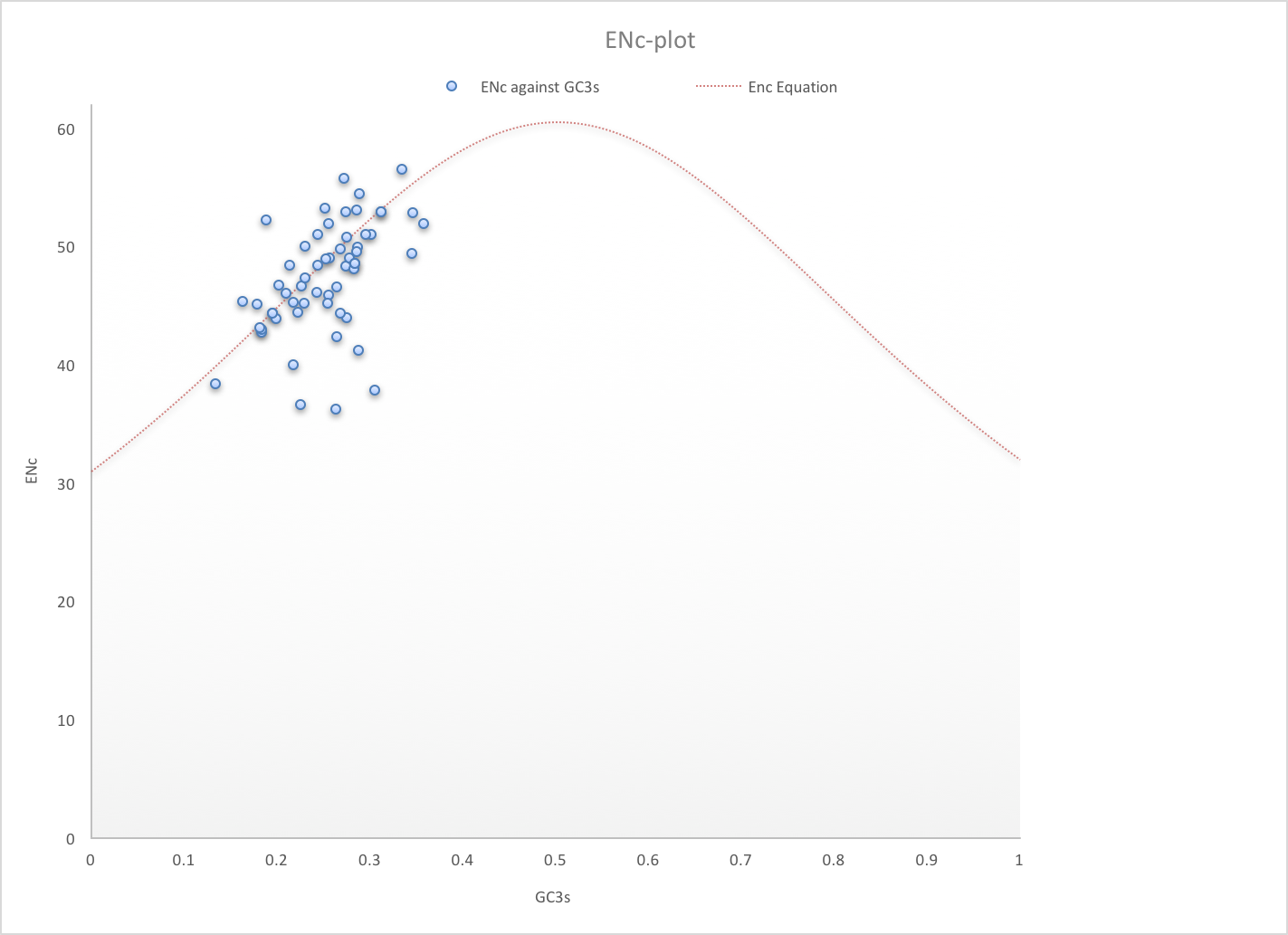

Supplement: FIGURE S2 — Estimation of the standard effective number of codon (Nc) was tabulated using the equation N(c) = 2 + s + 29/[s(2) + (1 – s)(2)], where s denotes GC3s (Wright, 1990). [file Image_2.PNG]

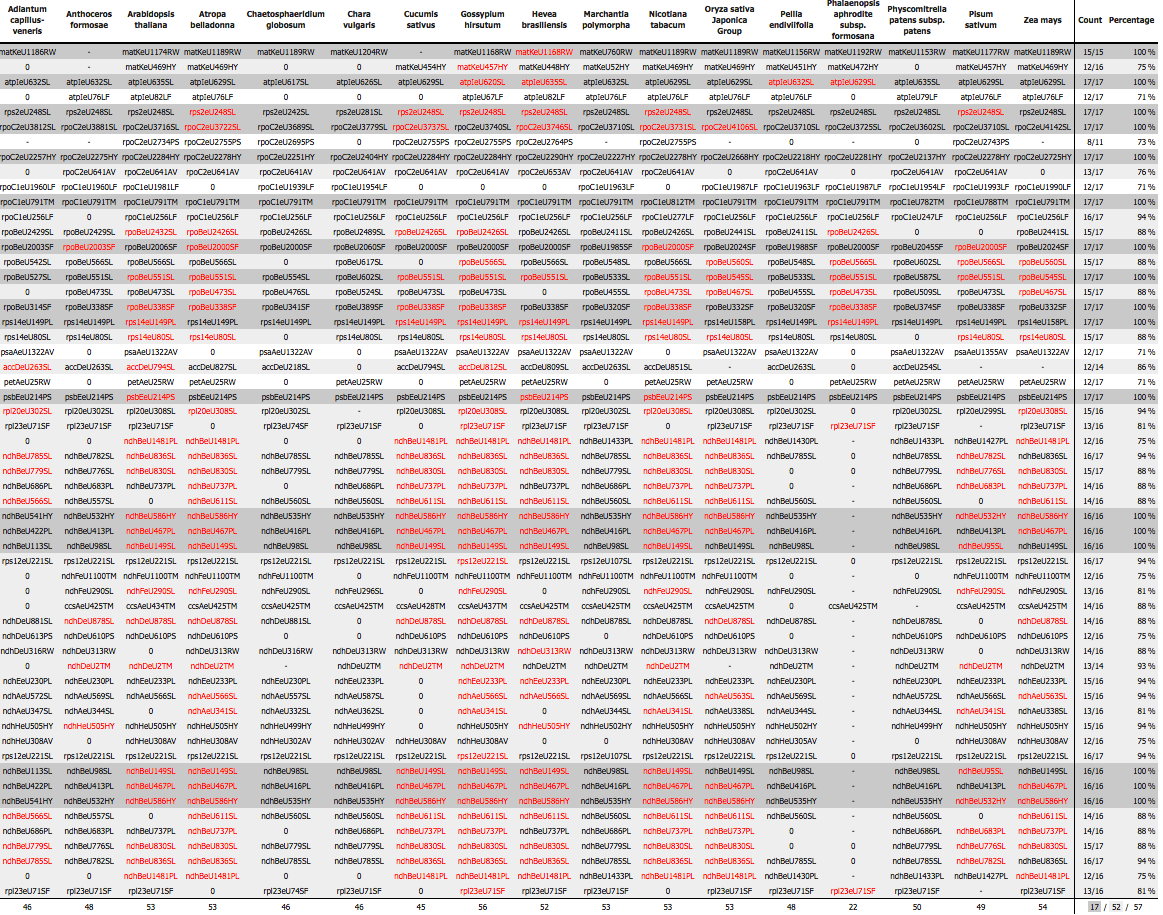

Supplement: FIGURE S3 — RNA editing output from PREPACT2 tool. [file Image_3.PNG]
