# Supplementary material for: Chloroplast Genome Analysis of Resurrection Tertiary Relict Haberlea rhodopensis Highlights Genes Important for Desiccation Stress Response
Source: Front Plant Sci. 2017 Feb 20;8:204. doi: 10.3389/fpls.2017.00204 (PMC5316520; doi:10.3389/fpls.2017.00204)

**Supplementary Data 1**

| **Results from QUAST analysis. QUAST was run on the contigs and scaffolds, obtained from the ABySS assembly and SSPACE scaffolding, respectively.** | | |
| --- | --- | --- |
|  | Post ABySS contigs | Post SSPACE scaffolds |
| Contigs | 123 | 71 |
| Largest contig | 14595 | 27644 |
| N50 | 8354 | 20267 |
| N75 | 3339 | 16015 |
| L50 | 6 | 3 |
| L75 | 13 | 5 |

**FastQC - Distributions of sample1 read lengths after merging by FLASH**
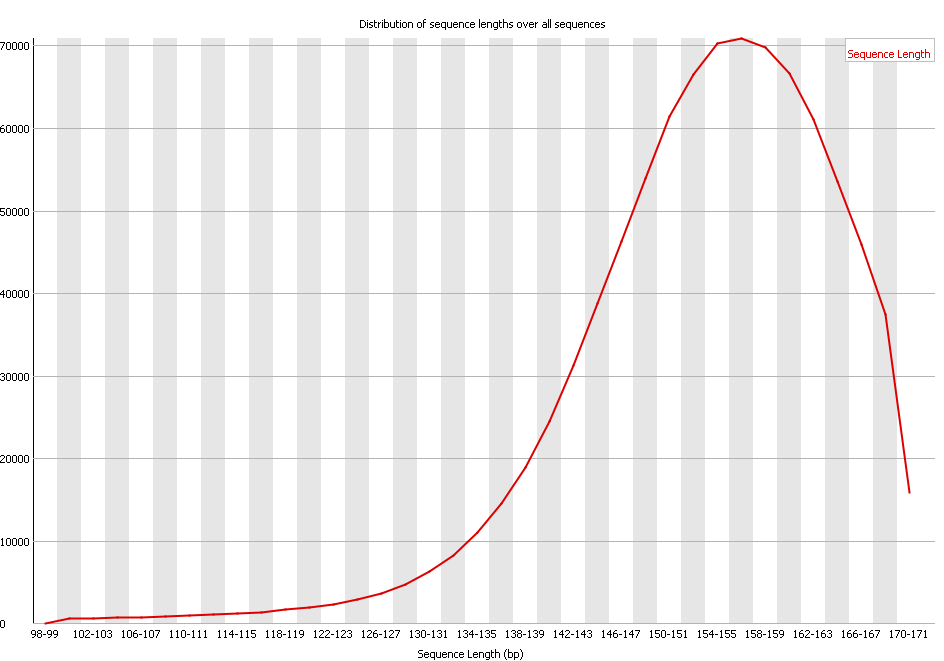


**FastQC - Quality control of sample1 merged reads**
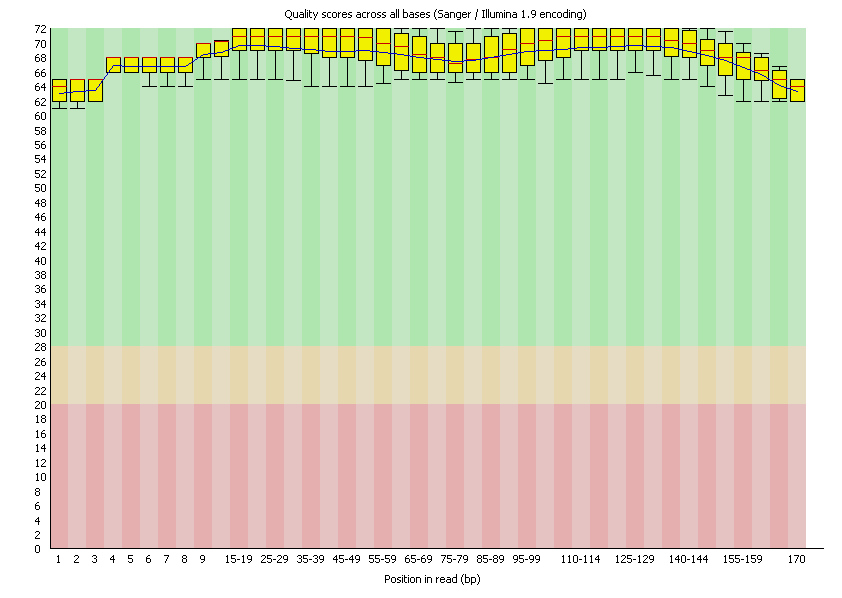


**FastQC - Distributions of sample2 read lengths after merging by FLASH**
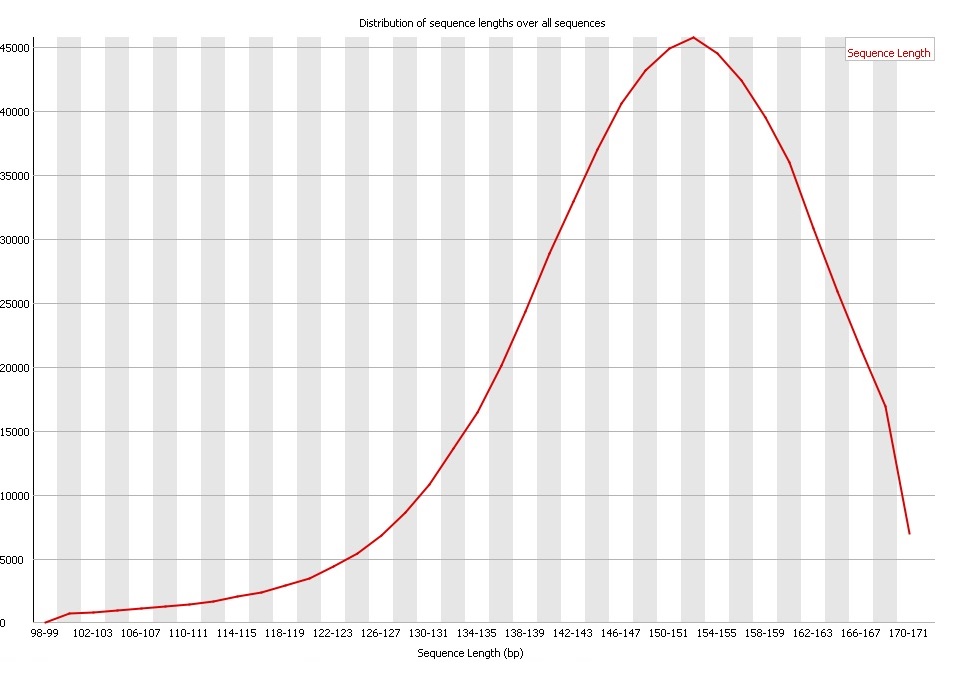


**FastQC - Quality control of sample2 merged reads**


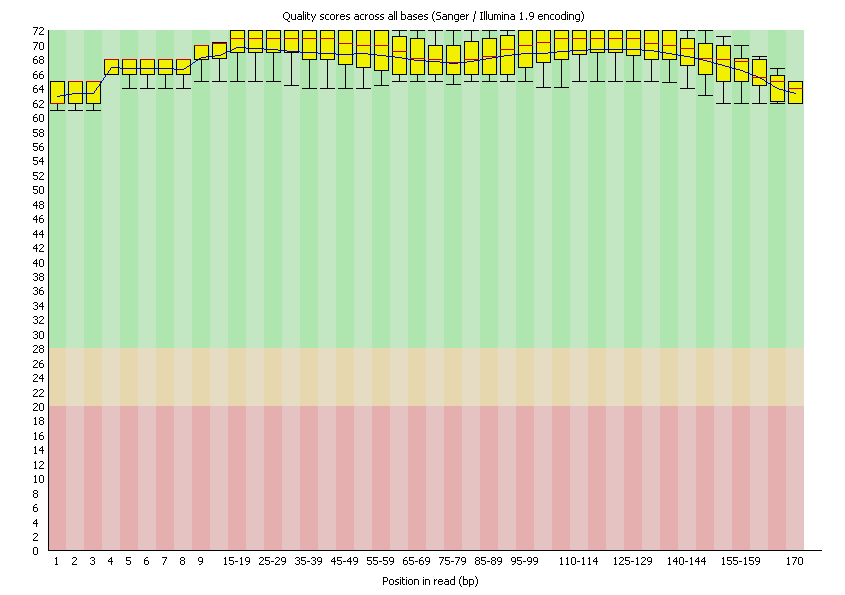

Supplement: DATA SHEET S1 — Merged read quality, length distribution and QUAST results. [file Data_Sheet_1.DOCX]
