# Supplementary material for: Chloroplast Genome Analysis of Resurrection Tertiary Relict Haberlea rhodopensis Highlights Genes Important for Desiccation Stress Response
Source: Front Plant Sci. 2017 Feb 20;8:204. doi: 10.3389/fpls.2017.00204 (PMC5316520; doi:10.3389/fpls.2017.00204)
Supplement: DATA SHEET S2 — IQtree consensus tree. [file Data_Sheet_2.DOCX]

Contree file content:

(Ageratina_adenophora:0.006216,((((((Anthriscus_cerefolium:0.010425,Daucus_carota:0.010397)100:0.027160,(Eleutherococcus_senticosus:0.003569,Panax_ginseng:0.005690)100:0.008733)100:0.013367,((Arabidopsis_thaliana:0.100466,Spinacia_oleracea:0.075379)100:0.019905,((((Atropa_belladonna:0.006215,(Datura_stramonium:0.005685,((Solanum_bulbocastanum:0.000721,Solanum_tuberosum:0.001465)99:0.000400,Solanum_lycopersicum:0.002874)100:0.006093)100:0.000751)100:0.001395,(((Nicotiana_sylvestris:0.000003,Nicotiana_tabacum:0.000067)100:0.001870,Nicotiana_undulata:0.002507)96:0.000397,Nicotiana_tomentosiformis:0.003865)100:0.002232)100:0.021776,Ipomoea_purpurea:0.056923)100:0.009908,((((Boea_hygrometrica:0.021461,Haberlea_rhodopensis:0.008336)100:0.018801,(Salvia_miltiorrhiza:0.037284,Sesamum_indicum:0.010427)100:0.003648)100:0.009208,(Jasminum_nudiflorum:0.029519,((((Olea_europaea:0.000403,Olea_europaea_europaea:0.000100)62:0.000026,Olea_europaea_maroccana:0.000041)66:0.000129,Olea_europaea_cuspidata:0.000349)100:0.001148,Olea_woodiana_woodiana:0.000919)100:0.005177)100:0.008210)100:0.012714,Coffea_arabica:0.049401)56:0.001305)100:0.010105)100:0.006119)100:0.008592,Trachelium_caeruleum:0.067059)100:0.031946,Lactuca_sativa:0.011599)100:0.002026,Jacobaea_vulgaris:0.017145)100:0.005482,(Guizotia_abyssinica:0.005073,Helianthus_annuus:0.007732)99:0.000775);
